# How to Puncture the Maxillary Antrum

**Published:** 1910-08-06

**Authors:** 


### Laryngology and Rhinology.

### HOW TO PUNCTURE THE MAXILLARY ANTRUM.

Lately Killian's method of puncture through the middle nasal meatus has been tried, but is probably inferior to the older intra-nasal method, in which the cavity is entered very near its lowest point. To puncture an antrum, then, there are required besides the lamp and head mirror, Lichtwitz's trocar and cannula, a Higginson's syringe provided with a small metal nozzle fitting accurately into the cannula, a quart glass measure, a nasal speculum and angled dressing forceps, a 1-gr. tabloid of cocaine, and a wisp of cotton-wool.

The cocaine is dissolved in 10 minims of tap water and the wool soaked in it and then packed round, and particularly up underneath, the anterior end of the inferior turbinate of the side suspected. After five minutes it is removed, and the point of the trocar, shielded by the slightly projecting end of the cannula, placed underneath the lower border of the inferior turbinate at a point three-quarters of an inch from its anterior extremity. The instrument is now directed outwards, backwards, and upwards. The two former directions come easily enough, but it is a little hard to get the necessary upward direction consistently with keeping the point below the turbinate body. Then work the trocar (the point being made to project again) "just like a brad-awl,"

but with a little less force. In nearly every case there is a sensation as of something suddenly giving, and a thin partition being penetrated. The trocar is now withdrawn, and the cannula remains firmly held, pointing more or less on a line drawn from the outer angle of one orbit to the opposite corner of the mouth. Now fit on the Higginson—empty—and cautiously pump air in. If you have made a false passage, air is much less likely to set up suppuration than lotion. If the cavity has been entered, no pain or resistance will be experienced by the patient and surgeon respectively. The Higginson is then unfitted and filled with warm half-strength boric lotion, which is injected while the patient holds the glass measure on his knees and bends his head well over to let the washings run into it from his nostril. These are to be examined for pus or flocculent debris. If a false passage has been made, which is quite unlikely if the above instructions have been carried out, the instrument must be withdrawn at once and another trial made.

